# Ectomycorrhizal *Cortinariaceae* species dominate class II peroxidase gene expression in a boreal forest soil

**DOI:** 10.1111/nph.71506

**Published:** 2026-08-13

**Authors:** Florian Barbi, Uwe Menzel, Domenico Simone, Tuula Niskanen, Björn D. Lindahl

**Affiliations:** ^1^ Laboratory of Microbial Ecology and Biogeography Institute of Microbiology of the Czech Academy of Sciences Videnska 1083 Prague 4 Czechia; ^2^ Department of Soil and Environment Swedish University of Agricultural Sciences Box 7014 SE‐75007 Uppsala Sweden; ^3^ Swedish University of Agricultural Sciences, SLUBI Box 7014 SE‐75007 Uppsala Sweden; ^4^ Botany and Mycology Unit, Finnish Museum of Natural History University of Helsinki Box 7 00014 Helsinki Finland

**Keywords:** boreal forest, ectomycorrhizal fungi, fungal traits, metatranscriptomics, organic matter decomposition and ecosystem fertility, saprotrophic Agaricomycetes

## Abstract

- Boreal forest ecosystems constitute a large terrestrial reservoir of carbon. In these nitrogen‐limited environments, release of nutrients through decomposition of soil organic matter is of fundamental importance. Fungi, particularly saprotrophic Agaricomycetes, are thought to drive this process using lignocellulolytic enzymes to degrade plant litter. However, some ectomycorrhizal fungal lineages have retained ancestral decomposition capabilities, yet evidence of their direct involvement in decomposition under field conditions is scarce.
- We used metatranscriptomics to examine the involvement of ectomycorrhizal fungi in the production of class II peroxidases in the soil of a Swedish boreal forest. We compared nutrient‐poor plots with more fertile ones and related the peroxidase‐expressing community to the total and cellulose‐degrading fungal communities.
- We found that overall expression of class II peroxidase genes was upregulated in nutrient‐poor soil, with ectomycorrhizal species in the *Cortinariaceae* family accounting for most of the transcripts. Among cellulose‐degrading fungi, there was a shift from saprotrophic Agaricomycetes in nutrient‐rich soil to dominance by Ascomycetes under nutrient‐poor conditions.
- Symbiosis may enable ectomycorrhizal fungi to use tree photoassimilates to drive energetically costly oxidation belowground. Ectomycorrhiza‐driven oxidation may, thereby, enable trees to indirectly regulate decomposition and nutrient cycling to maintain ecosystem productivity on unfertile soils.

## Graphical abstract

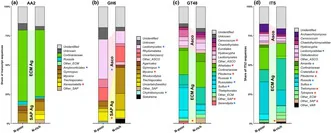

## Introduction

Boreal forests cover 12 million km^2^ of the Northern Hemisphere, representing a third of the world's forest area and constituting one of the most important terrestrial carbon pools, mainly due to the accumulation of organic carbon belowground (Pan *et al*., 2011; Harris *et al*., 2021). Moreover, the accumulation of a purely organic soil horizon above the mineral soil is associated with nitrogen retention (Kyaschenko *et al*., 2019) and low availability of inorganic nitrogen (Högberg *et al*., 2017). This makes boreal forests nitrogen‐limited ecosystems, in which the turnover of organic matter through decomposition is inextricably linked to soil fertility and ecosystem productivity (Clemmensen *et al*., 2013; Kyaschenko *et al*., 2019). In a context of uncertainty regarding the response of terrestrial carbon sinks to global changes (Melillo *et al*., 2017; Terrer *et al*., 2021), the fate of soil organic matter in these forests and the mechanisms underlying its turnover are of major concern.

Belowground organic matter accumulation in boreal forests does not seem to be controlled primarily by leaf litter input or short‐term litter decomposition rates. Rather, root‐associated fungi play a pivotal role, both in building up organic stocks and as regulators of decomposition (Clemmensen *et al*., 2013; Kyaschenko *et al*., 2017b, 2019; Lindahl *et al*., 2021). In particular, the activity of manganese peroxidases has been identified as a negative predictor of organic matter stocks (Kyaschenko *et al*., 2017b). Availability of manganese (Mn) has also been highlighted as a strong negative predictor of carbon (C) pools in the organic horizon in large‐scale inventories (Stendahl *et al*., 2017; Kranabetter, 2019), as well as in field experiments (Zhang *et al*., 2024). Manganese peroxidases belong to the class II peroxidases, which are a family of potent oxidative enzymes that evolved within the fungal class Agaricomycetes. They are keystone enzymes that enable degradation of lignin, mediated by highly oxidative Mn^3+^ ions, thereby exposing previously protected carbohydrate polymers (e.g. cellulose and hemicellulose) to enzymatic hydrolysis (Floudas *et al*., 2012; Kellner *et al*., 2014; Barbi *et al*., 2019; Mattila *et al*., 2022). In soils, manganese peroxidases have also been proposed to facilitate decomposition of a broader range of phenol‐rich substrates, including nitrogen‐containing fungal necromass rich in melanin, as well as condensed tannins bound to proteins or chitin (Fernandez *et al*., 2013; Adamczyk *et al*., 2017; Packard, 2026).

Fungi are the main decomposers of particulate organic matter, and especially, saprotrophic Agaricomycetes have a broad enzymatic potential to degrade the principal components of plant cell walls (Mycorrhizal Genomics Initiative Consortium *et al*., 2015; Hage & Rosso, 2021). Genes coding for hydrolytic extracellular enzymes, notably those involved in carbohydrate depolymerization, are common in microbial genomes, including those of fungal ascomycetes and basidiomycetes. However, some saprotrophic Agaricomycetes, known as white‐rot fungi, also possess genes coding for extracellular peroxidases, and their ability to efficiently oxidize lignin and other phenolics makes them particularly efficient decomposers of recalcitrant organic substrates (Sinsabaugh, 2010; Floudas *et al*., 2012). In a boreal forest context, there are indications that these white‐rot saprotrophs are favored by high nitrogen availability and a slightly higher pH (Sterkenburg *et al*., 2015; Lindahl *et al*., 2021). Efficient decomposition of recalcitrant organic substrates, in turn, increases turnover and availability of organic nitrogen, suggesting a positive feedback between forest fertility and decomposition rates (Vitousek & Howarth, 1991; Wardle *et al*., 2003) linked to the proliferation of saprotrophic Agaricomycetes in more fertile environments (Kyaschenko *et al*., 2017b, 2019). Ectomycorrhizal fungi have been proposed to hamper this decomposer feedback by competitive induction of nitrogen limitation in saprotrophs (Orwin *et al*., 2011) – the so‐called Gadgil hypothesis (Gadgil & Gadgil, 1971). Recent studies have suggested that the strength and direction of ectomycorrhizal–saprotroph interactions are strongly context‐dependent along gradients of nutrient availability and organic matter quality (Fernandez & See, 2025; Pena *et al*., 2025). Notably, Pena *et al*. (2025) not only observed a more apparent ‘Gadgil suppression’ of saprotrophs in relatively fertile temperate forest soils but also noted that under nitrogen poor conditions, certain ectomycorrhizal taxa, capable of mobilizing nitrogen from complex organic substrates, influenced saprotrophic Agaricomycetes negatively. This pattern is particularly relevant in the context of boreal forest soils, characterized by low pH and high organic matter carbon to nitrogen (C : N) ratios. Indeed, Kyaschenko *et al*. (2017b) observed that nitrogen availability, relative abundance of saprotrophic Agaricomycetes, and Mn peroxidase activity all decreased with increasing relative abundance of ectomycorrhizal fungi, while carbon stocks increased.

Among the ectomycorrhizal fungi, there is variation among taxa in the extent to which they have retained class II peroxidase encoding genes during evolution from saprotrophic ancestors (Bödeker *et al*., 2009; Miyauchi *et al*., 2020). The ability of these ectomycorrhizal fungi to oxidize organic matter and contribute to its biochemical transformation is increasingly acknowledged (Lindahl & Tunlid, 2015; Lindahl *et al*., 2021). High abundance of some species, especially from the family *Cortinariaceae* (previously including only one genus; *Cortinarius*, but currently divided into 10 genera (Liimatainen *et al*., 2022)), has been associated with peroxidase activity hotspots (Bödeker *et al*., 2014; Packard *et al*., 2025) and reduced stocks of organic matter (Lindahl *et al*., 2021). Furthermore, peroxidase activity correlated positively with ectomycorrhizal fungal species richness in a Californian pine forest (Talbot *et al*., 2013), and Mn‐peroxidase activity was linked to *Cortinariaceae* abundance in Swedish pine forests (Kyaschenko *et al*., 2017a). Thus, while the mechanistic link remains to be clarified, it has been proposed that these ‘ectomycorrhizal decomposers’ are nitrogen miners, which are able to access retained organic nitrogen by directly participating in breakdown of recalcitrant organic matter (Lindahl & Tunlid, 2015; Shah *et al*., 2016; Jörgensen *et al*., 2025; Packard, 2026). To understand the functioning of the boreal forest ecosystem, it is crucial to identify and uncover the precise roles of key actors in decomposition of organic matter, particularly those capable of producing extracellular peroxidases, and how they respond to nitrogen availability. On the one hand, a decrease in available nitrogen could increase the hampering effect of ectomycorrhizal fungi on saprotrophs (i.e. Gadgil suppression) and, thus, further reduce saprotrophic decomposition (Fernandez & Kennedy, 2016; Kyaschenko *et al*., 2017b). On the other hand, some ectomycorrhizal species may increase their own oxidative decomposition as nutrients become more scarce and trees allocate more carbon to ectomycorrhizal nitrogen‐mining (Defrenne *et al*., 2019; Argiroff *et al*., 2022; Jörgensen *et al*., 2024). These contrasting responses could differentially influence soil organic matter dynamics and may depend on the enzymatic strategies of the dominant ectomycorrhizal species and their interactions with saprotrophic fungi (Fernandez & See, 2025).

In general, many have attempted to predict ecosystem functions by analyzing microbial community composition (Jurburg & Salles, 2015; Graham *et al*., 2016), but community studies suffer from the lack of direct evidence on the ecological roles of described taxa. In contrast to prokaryotes, fungi have large genomes, and their gene contents are not necessarily reflected in expressed traits under natural conditions (Barbi *et al*., 2019). Moreover, measurements of enzyme activities in the field can only be linked to the responsible organisms by correlation (Talbot *et al*., 2013; Bödeker *et al*., 2014; Packard *et al*., 2025). Thus, the question remains open to which extent ectomycorrhizal fungi contribute actively and significantly to organic matter decomposition in boreal forest, and how they interplay with free‐living saprotrophic litter decomposers (Frey, 2019).

In this study, we used metatranscriptomics, specifically targeting gene expression of class II peroxidases, and related the peroxidase‐expressing community to the total and cellulose degrading fungal communities. Rather than describing the full complexity of fungal‐mediated decay processes, we aimed to conduce a focused analysis of genes coding for class II peroxidases that are pivotal for oxidation of complex phenolic compounds, as well as cellobiohydrolase genes pivotal for cellulose hydrolysis as an indicator of saprotrophic decomposition of plant litter. In addition, we targeted genes involved in β‐1,3‐glucan synthesis, playing a key role in fungal cell wall formation and hyphal growth, as a proxy for mycelial biomass production by the total fungal community. We also characterized the fungal communities by traditional DNA‐based internal transcribed spacer (ITS) barcoding, for reference. Metatranscriptomics emerged less than two decades ago as a method to study active taxonomic and functional diversity (Bailly *et al*., 2007; Damon *et al*., 2012), but its utility has been limited by a lack of available genomic resources and the low sequencing depth of previous technologies. Today, high‐throughput mRNA sequencing offers the possibility of assessing the functional role of forest soil microorganisms – that is who is doing what – by targeting transcription of specific genes, representative of ecophysiological traits, such as various aspects of organic matter decomposition, directly in the field and at ecologically relevant scales (Auer *et al*., 2024). Thus, in a Swedish old‐growth forest, where a negative correlation was previously observed between ecosystem fertility and belowground carbon storage (Kyaschenko *et al*., 2017b), we identified particularly N‐rich and N‐poor 8 × 8 m plots and sequenced RNAs from the organic horizon, assessing transcription of genes coding for:

1. Class II peroxidases (AA2) – a family including oxidative enzymes such as manganese‐, lignin‐, and versatile peroxidases associated with oxidative breakdown of complex phenolic macromolecules and identified as predictors of organic matter loss (Kellner *et al*., 2014; Stendahl *et al*., 2017; Kyaschenko *et al*., 2017b).
2. Cellobiohydrolases (GH6), active on cellulose chains and used as a marker of litter saprotrophism (Zhao *et al*., 2013; Barbi *et al*., 2019; Miyauchi *et al*., 2020).
3. 1,3‐β‐glucan synthase (GT48), generally associated with fungal growth (Hasby *et al*., 2021).
4. β‐tubulin (TUB), used as a proxy for overall fungal transcriptional effort to normalize AA2, GH6, and GT48 transcript abundances at the community level.

We tested the following hypotheses:

1. Transcription of class II peroxidases would be relatively lower in N‐poor plots (Kyaschenko *et al*., 2017b) linked to constrained proliferation of saprotrophic Agaricomycetes (i.e. a relatively lower GH6 expression in N‐poor plots and/or a lower share of saprotrophic Agaricomycetes in the GT48 and GH6 expressing community).
2. Ectomycorrhizal fungi, particularly in the genera from the family *Cortinariaceae*, would actively express genes coding for class II peroxidases, and their relative share of the transcripts would be higher in N‐poor plots (Bödeker *et al*., 2014; Argiroff *et al*., 2022).

## Materials and Methods

### Study site and soil sampling

**Fig. 1:**
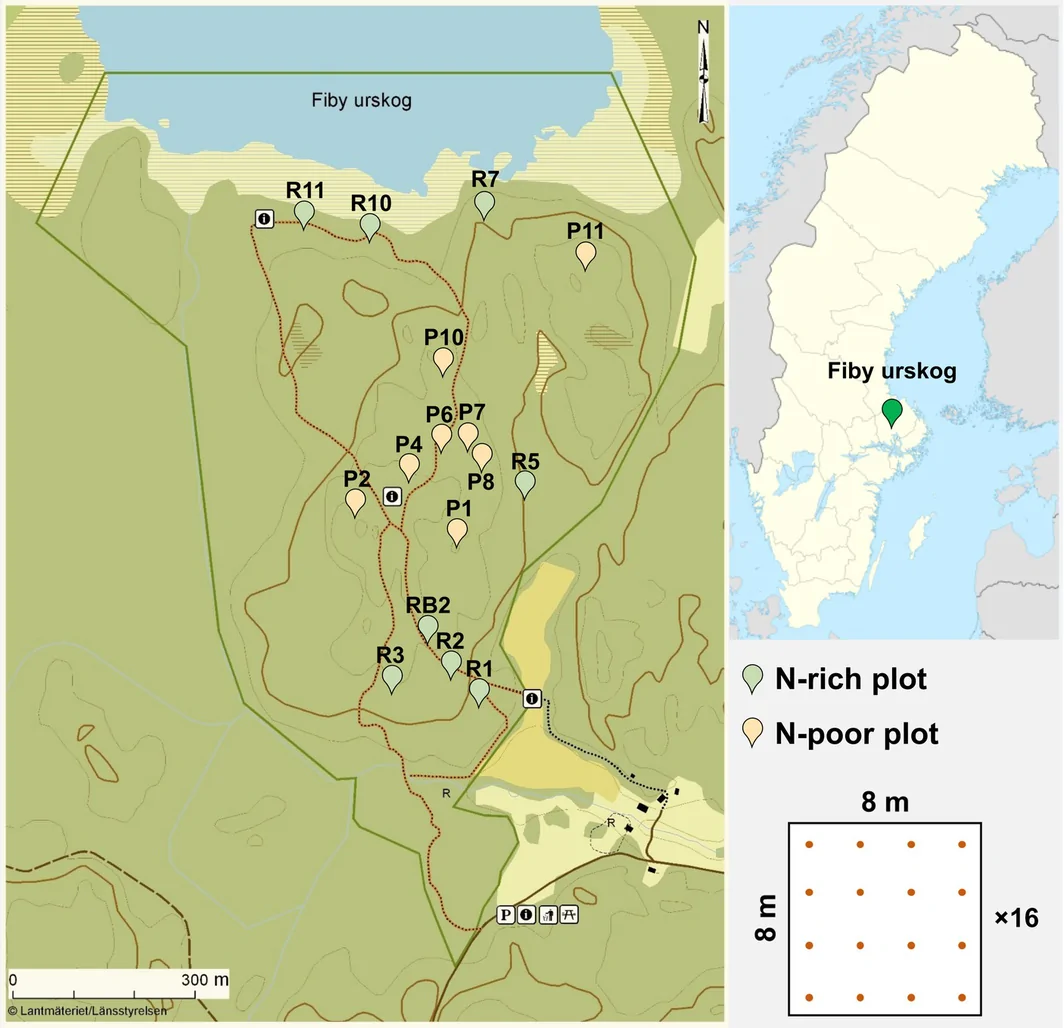
Map of sampling site, Fiby Urskog. Soil samples were collected from eight nitrogen (N)‐rich Norway spruce‐dominated plots (green) and eight Scots pine‐dominated N‐poor plots (yellow) at Fiby Urskog (Sweden). These composite samples consisted of 16 merged soil cores collected from the organic horizon within 8 × 8 m plots, during four sampling events, with four cores collected per plot per event. Characteristic of the plots are presented in Supporting Information Table S1.

The study was carried out in an old‐growth boreal forest left without major human intervention for several centuries – the 64‐hectare Fiby Urskog nature reserve, located 16 km west of Uppsala, Sweden. This forest is composed of a variety of forest types, characterized by different vegetation composition and soil and ecosystem fertility. The study was conducted in the same area as a previous study by Kyaschenko *et al*. (2017b), but we identified new plots to obtain a larger contrast between plots with high and low fertility. Soil cores were collected in 16 plots of 8 × 8 m. Eight plots were situated in high‐productive and nutrient‐rich areas (N‐rich) dominated by Norway spruce (*Picea abies* (L.) H. Karst), while eight others were situated in low‐productive and nutrient‐poor areas (N‐poor) dominated by Scots pine (*Pinus sylvestris* L.). More details about plot coordinates, characteristics, and vegetation are presented in Supporting Information Table S1. By necessity, the N‐poor plots were somewhat clustered at slightly higher elevation, but the N‐rich plots were dispersed, and we went as far as we could to obtain an even distribution (Fig. 1). The different dominant tree species in our sampling design largely reflected a natural fertility gradient, where pine commonly dominated in nutrient‐poor sites, and spruce‐dominated in nutrient‐rich sites (Shanin *et al*., 2014; Sterkenburg *et al*., 2015; Lukina *et al*., 2019). Soil cores of 3 cm diameter were collected from the entire depth of the organic horizon in autumn 2016 during four sampling events (on the 4^th^, 7^th^, 17^th^, and 27^th^ of October 2016). Four cores were collected from each plot per session for a total of 16 cores per plot at the end of the sampling period. The study focused on the humus layer, so live mosses, freshly deposited litter and mineral soil were quickly removed, after which each core was frozen within seconds on dry ice and thereafter stored at −80°C. Sampling was conducted during days with similar weather condition, and the order of the sampled plot was changed during each sampling event to minimize the potential effect of variation in gene expression during the day. Soil cores from each plot were pooled into composite samples to integrate gene expression across both time and space. The composite samples were homogenized by hand, freeze‐dried, and further stored at −80°C.

### mRNA and DNA extractions and sequencing

Total RNA was extracted following a cetyltrimethylammonium bromide (CTAB)‐based protocol with lithium chloride precipitation. Namely, 10 ml of freeze‐dried soil was ground manually in liquid nitrogen and mixed with 15 ml of extraction buffer containing 2% CTAB, 2% PVP, 100 mM Tris–HCl, 2 M NaCl, and 25 mM EDTA, along with 300 μl of β‐mercaptoethanol, and then incubated at 65°C for 10 min with frequent vortexing. Chloroform : isoamyl alcohol (24 : 1) was added to the mix, followed by vortexing and centrifugation. The resulting supernatant was mixed with 1/3 volume of 8 M LiCl and left to precipitate overnight at 4°C. The precipitate was then centrifuged, and the RNA pellet was dried, resuspended, and mixed with 1/10 volume of 3 M Na‐acetate and 2.5 volumes of cold absolute ethanol. The mix was left to precipitate overnight at −20°C. After centrifugation, the pellet was washed with 70% cold ethanol, dried at room temperature, and re‐suspended. Any residual DNA was eliminated using the AMPD1 DNase I kit (Sigma‐Aldrich). Finally, the extracted RNA was further purified using the RNeasy mini kit (Qiagen) following the manufacturer's instructions. All materials used during the RNA isolation procedure were either obtained RNase‐free from the manufacturer or subjected to autoclaving twice before use.

We depleted the samples in ribosomal RNAs by using magnetic beads provided with a 50 : 50 mix of probes from the RiboZero Human/mouse/rat and RiboZero Bacteria kits (Thermo Fisher Scientific, Waltham, MA, USA). Libraries for sequencing were prepared using the TruSeq stranded mRNA library preparation kit (Illumina, San Diego, CA, USA) and sequenced on the Illumina HiSeq2500 system, yielding 125‐bp paired‐end sequences. Library preparation and sequencing were performed by the SNP&SEQ Technology Platform of SciLifeLab, Uppsala, Sweden.

ITS2 markers were amplified, sequenced, and analyzed according to Clemmensen *et al*. (2016). DNA was extracted from 50 mg of freeze‐dried and grinded soil, using the NucleoSpin Soil kit (Macherey‐Nagel, Düren, Germany). PCR amplification with DreamTaq polymerase (Thermo Fisher Scientific) was performed in triplicates using the fungal‐specific gITS7 primer (Ihrmark *et al*., 2012) combined with ITS4(a) with both forward and reverse primers fitted with unique 8‐bp sample identification tags. The following cycling programme was used: 5 min at 95°C; 20–35 cycles of 30 s at 95°C, 30 s at 56°C, and 30 s at 72°C; 7 min at 72°C. Cycle numbers were adapted to obtain ‘weak but visible’ bands on the gel, as recommended in Castaño *et al*. (2020). PCR products were purified using the AMPure kit (Beckman Coulter, Indianapolis, IN, USA), DNA concentrations were established using a Qubit fluorometer (Life Technologies, Carlsbad, CA, USA), and DNA from each sample was pooled and purified again using the E.Z.N.A. Omega cycle pure kit (Omega Bio‐Tek, Norcross, GA, USA). The composite library was sequenced on the Sequel I platform (Pacific Biosciences, Menlo Park, CA, USA) by SciLifeLab NGI after the addition of sequencing adaptors by ligation.

### Targeted screening for gene families using a custom metatranscriptomics pipeline

Raw paired‐end reads were subjected to quality control using FastQC (Andrews, 2010), which indicated that between 91% and 97% of bases had a Q‐score above 30. Trimming of sequencing adaptors and removal of sequences containing low‐quality bases were performed with Trimmomatic, using default settings (Bolger *et al*., 2014). To study specific functional gene families, we developed a targeted approach to isolate specific reads from the metatranscriptome: RING: Reads Isolator from Nucleotidic Giga‐dataset (https://github.com/domenico‐simone/RING).

Briefly, Hidden Markov Models (HMMs) were designed based on a large number of reference sequences obtained from the JGI Mycocosm database (DOE Joint Genome Institute, Berkeley, CA, USA, https://mycocosm.jgi.doe.gov/mycocosm/home). The reference sequences for AA2 family were selected from Basidiomycetes using an ‘AA2’ query and filtered by ‘User Annotations’. A similar procedure was applied for GH6 and GT48 family references, but selections included all Dikarya. References for β‐tubulin (TUB) coding genes were selected using the search query ‘IPR002453 AND KOG1375’ across all Dikarya. The reference sequences were aligned using ClustalW (Thompson *et al*., 1994), and a maximum likelihood phylogeny was constructed using ‘PhyML’ (Guindon *et al*., 2010). The phylogenetic tree was then split into subtrees (one to three) at basal branches, and each subtree was further subdivided into subgroups. The number of sub‐groups was equal to 30% of the number of reference sequences included in the associated sub‐tree (e.g. a subtree containing 60 sequences would be subdivided in 18 subgroups). To obtain a phylogenetically balanced selection of references, a single sequence was picked randomly from each subgroup, with sampling repeated 10 times to ensure consistency. HMMs were constructed based on the sampled reference sequences, using the ‘hmmbuild’ command in HMMER (http://hmmer.org; Eddy, 2011). Then, the 10 HMMs from each subtree were used to extract homolog sequences from the filtered metatranscriptome reads, using the ‘nhmmer’ command. The results of these 10 trials were merged to obtain a pool of reads extracted from each subtree. Finally, the outputs of the different subtrees were compared in a Venn diagram to evaluate generality/specificity of the models and to choose the optimal number of subtrees for further analyses, aiming for a full coverage of gene families.

Extracted sequences were cleaned from sequences containing stop codons, using TransDecoder (https://github.com/TransDecoder). Contig assembly was performed with SPAdes (Bankevich *et al*., 2012), using default parameters with ‐‐rna flag for all samples together. Contigs with a coding region of at least 279 bp were identified, using TransDecoder, to get rid of single‐read contigs and identify reading frames and direction. All contigs were aligned pairwise, using BLASTm, and contigs that were > 97% similar to longer contigs were deleted.

The filtered extracted sequences from each sample were then mapped back against the contigs using Bowtie2 (Langmead & Salzberg, 2012) and filtered with SAMtools (Li *et al*., 2009) based on mapping quality score, using the –q option. Low ‐q values (min = 0) retain more reads by allowing lower mapping confidence, whereas higher values (max = 42) are more conservative, keeping only high‐confidence mappings. Being too conservative risks losing valuable information, while allowing ambiguous mappings could artificially inflate the relative abundance of contigs with conserved regions. To balance these issues, we tested different ‐q values for each gene family and selected the ones where the number of filtered mapped reads was closest to the total number of extracted sequences. This does not imply that each read was mapped only once but represents a compromise to minimize both types of bias. The output was sorted, indexed, and converted to count tables by SAMtools. The contigs were translated to amino acid sequences in MEGA (Tamura *et al*., 2021) and subjected to BLAST searches against relevant enzyme families in the CAZy database (Drula *et al*., 2022) to filter out nontarget contigs (Tables [Link], [Link]). The TUB contigs were subjected to BLAST searches against Swiss‐Prot and Uniref90 databases, and only those with best matches to fungal TUB genes were retained. Finally, total transcript abundances of the targeted families were normalized against fungal TUB transcript abundance to estimate relative transcriptional investment at the level of fungal communities. The resulting expression ratios were log_10_‐transformed to meet the normality assumption, and Student's *t*‐tests were then used to compare expression ratios between plot types.

The total numbers of extracted reads were 242 446 for AA2, 47 840 for GH6, and 434 978 for GT48. From these, 729 AA2, 490 GH6, and 2398 GT48 contigs were assembled. After open reading frame filtering, clustering, and removal of nonfungal and nontarget families, we obtained 577 AA2, 288 GH6, and 1898 GT48 contigs, with average coverages of 61×, 20×, and 20×, respectively (Table 1). Nontargeted contigs (discarded) included plant haem peroxidases, cytochrome C peroxidases, and various nonclass II or unidentified peroxidases for AA2 (Table S2), nonfungal (bacterial or other eukaryotic) cellobiohydrolases/cellulases/glucanases for GH6 (Table S3), and plant callose synthases for GT48 (Table S4). Finally, for identification, we selected the most expressed contigs, resulting in a total of 146 AA2, 63 GH6, and 267 GT48 contigs, having an average coverage of 200×, 67×, and 92×, respectively (Table 1).

**Table 1:** Summary of the RING pipeline output, contigs assembly, and processing for the studied gene families: class II peroxidases (AA2), cellobiohydrolases (GH6), glucan synthases (GT48), and for β‐tubulin (TUB).

Gene family	AA2	GH6	GT48	TUB
Extracted sequence (HMMs)	242446	47840	434978	638028
Assembled contigs	729	490	2398	4215
Filtered contigs after ORFs screening	704	417	2051	3500
Contigs after clustering (97%)	657	412	1909	3318
No. of mapped reads (samtools Q value)	237 605 (19)	50 150 (1)	430 598 (22)	1182 859 (42)
All contigs mean coverage	61	20	20	101
All contigs median coverage	10	6	6	17
No. of contigs after BlastX Removal of nonfungal and nontargeted families	577	288	1898	640
Most expressed contigs (80% of the mapped reads)	146	63	267	NA
Most expressed contigs mean coverage	200	67	92	NA
Most expressed contigs median coverage	105	42	26	NA

### Metabarcoding analyses

ITS amplicon sequences were filtered and clustered using the SCATA pipeline (scata.mykopat.slu.se; Ihrmark *et al*., 2012). Sequences were quality checked to remove those shorter than 100 bp, with mean quality scores lower than 20, with individual bases with a quality score lower than 3, or with a missing 3′ or 5′ tag. Remaining sequences were screened for the primers, requiring a minimum match of 90%, and reverse complemented, if necessary. After removal of primer sequences and sample tags, sequences were clustered through pairwise comparisons using USEARCH (Edgar, 2010) followed by single linkage clustering, with the minimum similarity to the closest neighbor required to enter a cluster set at 98.5% and equal penalties for insertions and substitutions. Homopolymers were collapsed to 3 bp during clustering, and globally unique genotypes were removed to reduce the incidence of sequencing errors. Representative ITS2 sequences from the most abundant clusters, which accounted for 80% of the total amplicon sequences in N‐poor and/or N‐rich conditions, were identified against the UNITE database (Abarenkov *et al*., 2024), and clusters were aggregated at the genus level (Table S6).

A total of 139 226 ITS sequences passed the quality filter and were clustered into 1084 operational taxonomic units (OTUs), of which 1040 were identified as fungal. For subsequent analyses, we selected the mRNA contigs/ITS OTUs accounting for 80% of the total count in N‐poor and/or N‐rich conditions (Table S6).

### Phylogenetic analyses

The most expressed contigs for AA2, GH6, and GT48 families, which accounted for 80% of the total expression of a gene family in N‐poor and/or N‐rich conditions, were subjected to phylogenetic analysis (Tables [Link], [Link]). Reference sequences were collected from JGI Mycocosm unrestricted data using BlastX, including the best reference match for every contig. Additional reference data from *Cortinarius alces*, *C. bovarius*, *C. glandicolor*, and *C. neofurvolaesus* (European Nucleotide Archive, Study ID PRJEB49625) and C. *ominosus* (BioProject PRJNA791499) from Liimatainen *et al*. (2022) were also included, as well as newly produced data from *C. caperatus* (BioProject PRJNA1277778). In total, 74, 106, and 43 unique reference sequences from identified material were included in the AA2, GT48, and GH6 phylogenies, respectively. Reference sequences were curated manually, after which sample and reference amino acid sequences were aligned together using MAFFT (Rozewicki *et al*., 2019). Phylogenetic trees were constructed by the maximum likelihood method, using RAxML with default parameters (Stamatakis, 2014). We accepted a bootstrap threshold of 75% to identify clades containing reference sequences with a consistent taxonomy (Fig. S1).

The expression of these most expressed contigs was summed per genus (or at a higher taxonomic level in some cases) on a per sample basis, and the average of samples of the same condition (N‐poor vs N‐rich) was calculated to estimate the proportion of contigs originating from different fungal genera (Tables [Link], [Link]). Nonmetric multidimensional scaling (NMDS) ordinations based on Bray–Curtis dissimilarity index of relative expression of the most expressed contigs, beta dispersion ‘betadisp’, and permanova ‘adonis2’ (method = ‘bray’, permutations = 999) were used to test the effect of the fertility condition on composition of the expressed genes. Wilcoxon tests were used to test for differences in relative abundance of taxa and guilds between plot types. All statistical analyses were performed in R v.4.4.1 (R Core Team, 2022).

### Soil analyses

We measured the content of total nitrogen (N) and carbon (C) using a TruMac analyzer (LECO, St. Joseph, MA, USA). Ammonium nitrogen (NH_4_^+^‐N) concentrations (mg kg^−1^) were measured using an Autoanalyzer AA3 (SEAL Analytical, Southampton, UK). Manganese (Mn) was extracted with 1 M ammonium acetate and measured using an Avio 200 ICP‐OES spectrometer (PerkinElmer, Shelton, CT, USA). Soil pH was measured in deionized H_2_O using a Multilab 540 pH instrument (WTW, Washington, DC, USA). Student's *t*‐tests were used to compare pH, extractable Mn, C : N, and C : NH_4_^+^‐N ratios between N‐poor and N‐rich plots.

## Results

Soil analyses indicated clear differences in chemistry between the two conditions. Soil pH was significantly higher (*P* < 0.001) in N‐rich (4.29 ± 0.06 (SE)) than in N‐poor (3.71 ± 0.03) conditions. Concentrations of extractable Mn were six times higher (*P* < 0.001) in N‐rich conditions. Conversely, the C : N and C : NH_4_^+^‐N ratios were significantly higher in N‐poor plots (*P* < 0.001), by factors of 1.2 and 1.8, respectively (Table S1).

A total of 16 samples were sequenced with an average of *c*. 125 M sequences per sample for a total metatranscriptome of *c*. 2 billion reads. We randomly subsampled 10 times 100 reads from each sample and performed a blastX to assess the overall profile of the metatranscriptome. An average of *c*. 72% had a positive hit, representing putative mRNA. Among these positive hits, we estimated that an average of 16% of the reads originated from Fungi, 14% from Bacteria, 10% from Plantae, 1.7% from Animalia, and < 1% from other Eukaryota, Archaea, and Virus (Table S7).

**Fig. 2:**
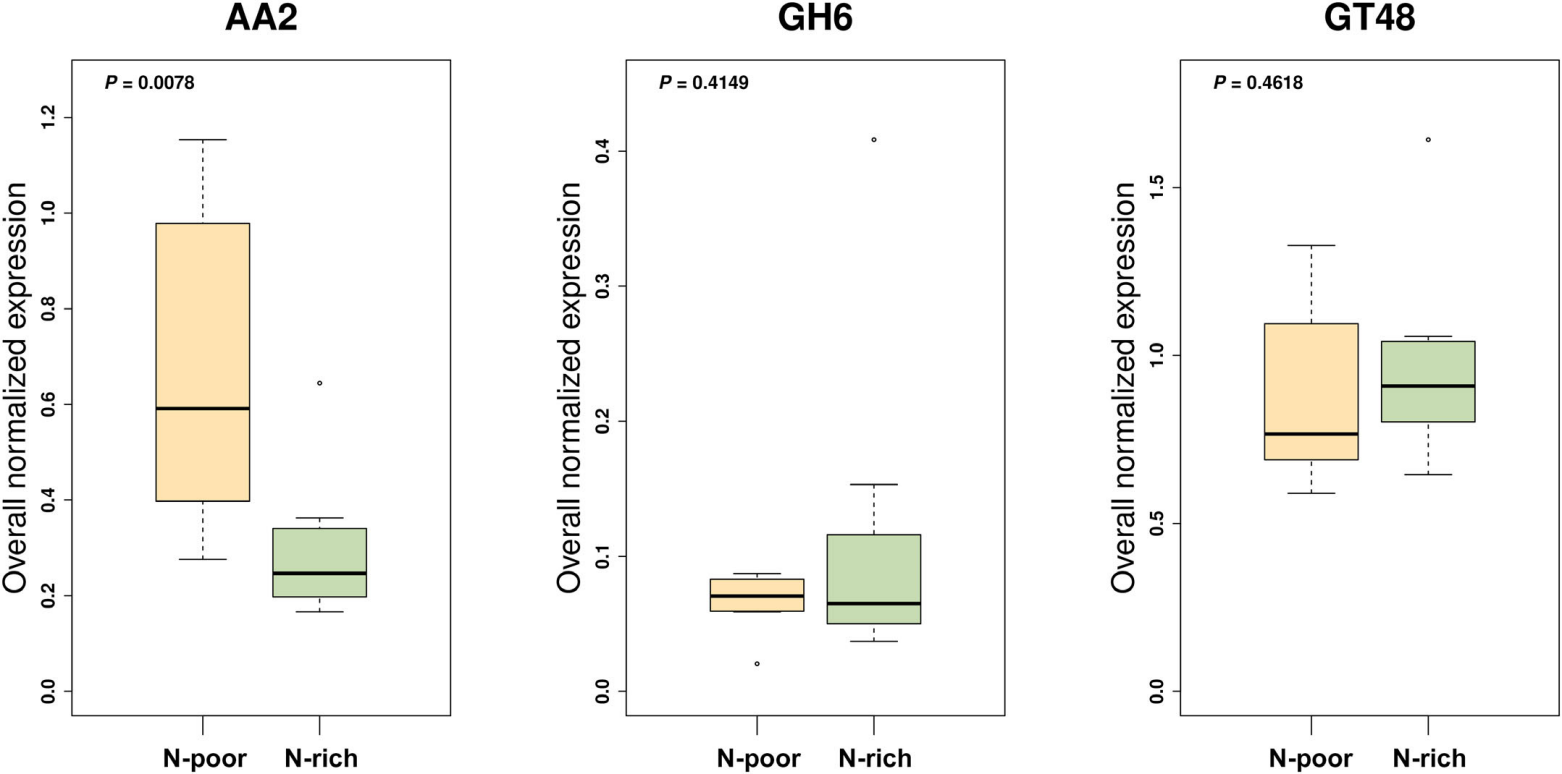
Overall normalized expression. Boxplots of overall expression (i.e. total reads mapped to all fungal contigs from a gene family) normalized against β‐tubulin (TUB) representing the global fungal transcriptional effort in production of class II peroxidases (AA2), production of cellobiohydrolases (GH6), and production of 1,3‐β‐glucan (GT48) under nitrogen (N)‐rich (green) and N‐poor (yellow) conditions. Error bars represent SD. Student's *t*‐tests (*P* < 0.05) were then used to compare expression ratios between plot types.

The overall AA2 (encoding class II peroxidases) expression was more than twofold higher (*P* = 0.007) in N‐poor (0.67 ± 0.12) than in N‐rich conditions (0.30 ± 0.06), while GH6 (encoding cellobiohydrolases) and GT48 (encoding β‐1,3 glucan synthase) expressions were similar in both forest types (Fig. 2).

**Fig. 3:**
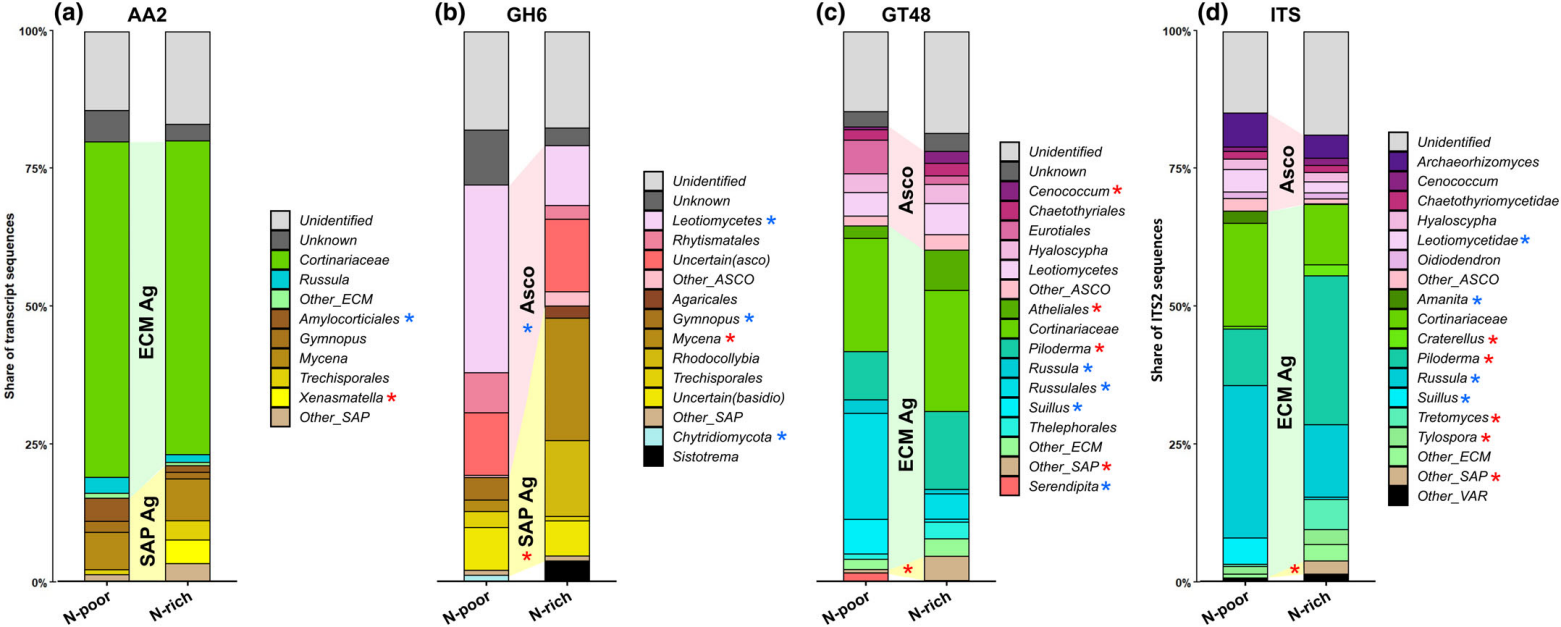
Share of transcripts and ITS2 sequence for each gene families among identified fungal contigs and OTUs. Stacked plots represent the proportion of the most expressed contigs/OTUs (i.e. accounting for 80% of the total relative expression/abundance of a gene family in nitrogen (N)‐poor and/or N‐rich conditions) among different fungal taxa. For each gene family, (a) Class II peroxidases – AA2, (b) cellobiohydrolases – GH6, (c) glucan synthases – GT48, and (d) for fungal OTUs – ITS, asterisks indicate significantly (Wilcoxon‐test *P* < 0.05) higher proportions for a fungal taxon or group (ECM Ag: ectomycorrhizal Agaricomycetes; Asco: ascomycetes; SAP Ag: saprotrophic Agaricomycetes) under N‐poor (blue stars) and N‐rich (red stars) conditions, taxa that does not belong to these groups were classified in various (VAR). Taxa with a relative abundance inferior to 2% are grouped in the ‘Other’ categories.

**Fig. 4:**
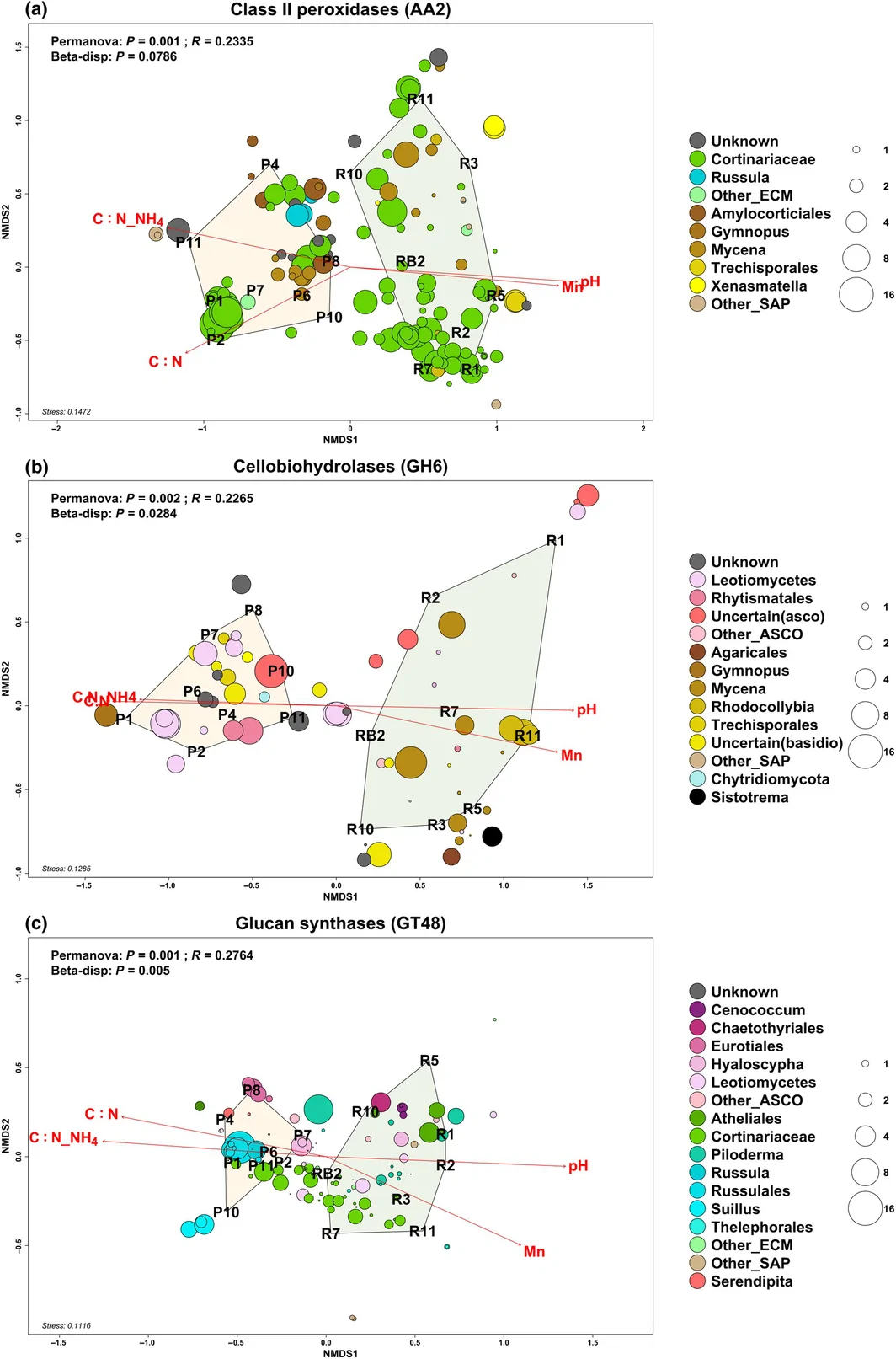
Nonmetric multidimensional scaling (NMDS) ordination of functional expressed genes across nitrogen (N)‐rich and N‐poor conditions. NMDS calculated using all fungal contigs of the different studied gene family: (a) class II peroxidases – AA2; (b) cellobiohydrolases – GH6; (c) glucan synthases – GT48, but only the most expressed contigs (i.e. accounting for 80% of the total relative expression of a gene family in N‐poor and/or N‐rich conditions) are displayed as symbols. Symbol area represents proportional relative expression of contigs across all samples and are colored according to identified fungal taxa. Red vectors represent fitted soil pH, concentration of extractable manganese (Mn), carbon‐to‐nitrogen ratio (C : N), and carbon to ammonium nitrogen (C : N_NH_4_). Polygons highlights group of samples from N‐rich (green) and N‐poor (yellow) conditions.

The GT48 contigs originated primarily from basidiomycetes under both N‐rich and N‐poor conditions (N‐rich: 71% of the share of transcript sequences reads that mapped to identified contigs, N‐poor: 74%) and second from ascomycetes (*c*. 23% for both conditions) (Fig. 3c) with distinct community composition between N‐rich and N‐poor conditions (Fig. 4c). Beta diversity of GT48 expression was significantly lower under N‐poor conditions. Overall, the largest proportion of sequences was ascribed to the ectomycorrhizal family *Cortinariaceae* (N‐rich: 28%, N‐poor: 25%), *Piloderma* (N‐rich: 18%, N‐Poor: 11%), *Russulales* (N‐rich: 5.9%, N‐Poor: 23%), *Russula* (N‐rich: 1.1%, N‐Poor: 2.9%), *Suillus* (N‐rich: 0.7%, N‐poor: 7.6%), and *Thelephora* (N‐rich 2.3%, N‐poor: 0%) as well as to the ascomycetous order *Helotiales*, including *Acephala* and *Hyaloscypha* (N‐rich: 4.8, N‐poor: 4.5), and other Letiomycetes (N‐rich: 7.3%, N‐poor: 5.2%) or to *Eurotiales* (N‐rich: 2.1%, N‐poor: 7.3%). Thus, the main differences were the low shares of *Russula/Russulales* and *Suillus* GT48 transcripts in the N‐rich spruce plots compared with the N‐poor pine plots, and twice as high proportion of *Piloderma* transcripts in N‐rich plots (Table S4). Interestingly, saprotrophic Agaricomycetes (mainly *Mycena* and *Rhodocollybia*) accounted for a small proportion of the GT48 transcripts in N‐rich plots (1.3% and 2.1%, respectively) and were almost absent among the GT48 transcripts from N‐poor plots.

The fungal community as assessed based on ITS sequences reflected the total active community described by GT48 transcripts (Fig. 3d). Ectomycorrhizal fungi dominated (*c*. 76%), followed by ascomycetes (N‐rich: 19%, N‐poor: 15%), while saprotrophic Agaricomycetes represented less than 2% and 0.1% of the identified contigs in the N‐poor and N‐poor plots, respectively (Table S6). The ectomycorrhizal fungal community was dominated by *Cortinariaceae*, *Russula*, and *Piloderma*. Although *Cortinariaceae* ITS sequences were more abundant in the N‐poor plots (N‐rich: 22%, N‐poor: 14%), the relative ITS proportions of the main ectomycorrhizal genera followed similar patterns as for GT48. Thus, *Russula* and *Suillius* were relatively more abundant in N‐poor plots, while *Piloderma*, *Tretomyces*, and *Tylospora* (which belong to the *Atheliales* observed in GT48) dominated in N‐rich plots. One notable difference was the significant presence of *Archaeorhizomyces* under both conditions, whereas this genus was absent from the active community based on GT48. However, it is not possible to determine whether this discrepancy results from the inability to identify GT48 within this genus due to a lack of references, or whether the detected ITS DNA originated from inactive fungi without significant GT48 expression.

Sequences mapping to identified AA2 contigs were equally distributed among fungal genera under N‐rich and N‐poor conditions (Fig. 3a). However, at the level of individual contigs, the different plot types had distinct AA2 expression patterns (Fig. 4a). On average, 78% of the AA2 transcripts originated from ectomycorrhizal fungal genera, mostly from the family *Cortinariaceae* (N‐rich: 70%, N‐poor: 73%). Lower levels of AA2 expression (i.e. a share of contigs < 5%) were also detected in the ectomycorrhizal genera *Russula*, *Hebeloma*, and *Hysterangium*. Saprotrophic fungal genera represented on average 25% of the transcripts, dominated by *Mycena* (N‐rich: 10%, N‐poor: 9.5%; Fig. 3a). Other saprotrophic AA2 contigs originated from Amylocorticiales, Trechisporales, *Gymnopus*, *Resinicium*, *Xenasmatella*, *Rhodocollybia*, Auriculariales, and Polyporales. Finally, species from the genus *Piloderma* are commonly expected to express AA2 (Argiroff *et al*., 2022; Jörgensen *et al*., 2024) but were absent among the most expressed contigs. Therefore, we checked the full AA2 blastx output against NCBI ‘nr’ and JGI Mycosom ‘Gene Catalog protein’ databases and found AA2 contigs matching with *Piloderma* references (i.e. AA2 contigs 185, 410, and 573 with > 93% similarily), but they represented only 0.084% of the AA2 transcripts (Table S2), which seems negligible in relation to the large share of GT48 expression ascribed to *Piloderma*.

Finally, we observed a distinct switch in the phylogenetic origin of GH6 expression between the two forest types (Fig. 3b), reflected in the distinct composition of GH6 transcripts between N‐rich and N‐poor conditions (Fig. 4b). In N‐rich plots, 63% of the sequences mapping to identified GH6 contigs originated from saprotrophic Agaricomycetes, with 29% from *Mycena*, and 15% from *Rhodocollybia*. In N‐poor plots, however, basidiomycetes only accounted for 25% of the transcripts, whereas most of the GH6 transcripts in N‐poor plots originated from ascomycetes (73%), with a dominance of Leotiomycetes (45%). As for GT48, beta diversity of GH6 expression was also significantly lower under N‐poor conditions.

## Discussion

### The pivotal role of ‘ectomycorrhizal decomposers’ in boreal forests

Consistent with our expectations, we found evidence suggesting a constrained proliferation of saprotrophic Agaricomycetes in N‐poor conditions. The saprotrophic Agaricomycetes' share of AA2 transcripts was similar under both fertility conditions, but their share of GT48 transcripts decreased dramatically under N‐poor conditions, consistent with patterns of total fungal community composition based on ITS data. Furthermore, although overall GH6 expression, reflecting investment in cellobiohydrolase activity, which is a key component of saprotrophic cellulose decomposition, did not differ between conditions, we observed a notable shift in the origin of GH6 transcripts, from saprotrophic Agaricomycetes under N‐rich conditions to ascomycetes in N‐poor plots. This pattern is consistent with previous observations of reduced abundance of saprotrophic Agaricomycetes in strongly nitrogen‐limited environments (Sterkenburg *et al*., 2015; Lindahl *et al*., 2021). In addition, GH6 beta diversity was clearly lower under N‐poor conditions. While GH6 reflects only one component of saprotrophic litter decomposition, this pattern may indicate strong environmental filtering of the fungal cellulose degrading community. Our observations are in line with the idea that constrained activity of saprotrophic Agaricomycetes in unfertile environments may be described as an amplifying feedback: unfavorable conditions (e.g. low pH, poor litter quality, and low Mn availability) restrict growth and activity of saprotrophic Agaricomycetes, thereby reducing plant nutrient availability by nonmycorrhizal pathways. This favors plant species with resource conservative traits linked to low litter decomposability (Wardle *et al*., 2003; Cornwell *et al*., 2008). The recalcitrant litter, in turn, may call for costly adaptations in saprotrophic fungi, reducing their growth and decomposer activity (Manzoni *et al*., 2012; Chakrawal *et al*., 2024). Intensified nitrogen limitation of both plants and saprotrophs could push the ecosystem toward a limit, at which the energy that saprotrophs can obtain from increasingly recalcitrant litter does not suffice to power decomposition (Sterkenburg *et al*., 2018). This situation might be aggravated by competition for nitrogen with mycorrhizal fungi (Orwin *et al*., 2011; Kyaschenko *et al*., 2017a) and, in a long‐term perspective, lead to ecosystem retrogression (Clemmensen *et al*., 2015).

In contrast to our first hypothesis, despite a lower proportion of saprotrophic Agaricomycetes in N‐poor plots, the overall relative transcription of fungal class II peroxidases rather increased under low fertility conditions, suggesting that under severe nitrogen limitation, certain ectomycorrhizal fungi allocate more resources to mobilization of tightly bound N (Packard, 2026). Interestingly, a sixfold lower Mn availability under nutrient‐poor conditions may suggests that a higher production of Mn peroxidases could be required to maintain a sufficient pool of oxidized Mn^3+^. In line with our second hypothesis, production of class II peroxidases under nitrogen limitation was driven primarily by ectomycorrhizal fungi. Unlike saprotrophic Agaricomycetes, ‘ectomycorrhizal decomposers’ fungi do not rely on lignin breakdown to access cellulose as a carbon source but are instead supported with high‐quality carbon by the trees and, supposedly, make use of their decomposer capabilities primarily to extract nitrogen and other nutrients bound in recalcitrant organic matter (Lindahl & Tunlid, 2015; Packard, 2026). Thereby, they are not vulnerable to environmental constraints on saprotrophic carbon acquisition. The roles and interactions of ectomycorrhizal and saprotrophic fungi in the decomposition process have been widely discussed (Averill & Hawkes, 2016; Fernandez & Kennedy, 2016; Shah *et al*., 2016; Kyaschenko *et al*., 2017a; Smith & Wan, 2019; Argiroff *et al*., 2022; Fernandez & See, 2025; Pena *et al*., 2025). In recent studies, the presence of certain ectomycorrhizal fungi was associated with low soil organic matter stocks, and ectomycorrhizal fungi were highlighted as essential in the decomposition process (Clemmensen *et al*., 2015, 2021; Lindahl *et al*., 2021). Our results support an important contribution of ‘ectomycorrhizal decomposers’ to oxidation of organic matter in boreal forest soils. We propose that the dominance of ectomycorrhizal fungi in the class II peroxidases expressing community could be a crucial feature of the boreal forests to maintain oxidative release of nutrients in unfertile soils. In this study, ectomycorrhizal fungi dominated class II peroxidases expression also in the N‐rich plots (albeit at a lower expression rate), but in even more fertile ecosystems, for example with arbuscular mycorrhizal (AM) trees (Phillips *et al*., 2013) or subjected to nitrogen deposition (Jörgensen *et al*., 2024), ectomycorrhizal decomposers tend to decline/disappear, and saprotrophs dominate decomposition. A dominant contribution of ectomycorrhizal species to peroxidase activity in the organic topsoil implies that this biochemical activity, which is central for organic matter decomposition, may be closely linked to tree carbon allocation belowground. When limited by low nutrient availability, trees may increase organic matter turnover by providing sugars to their associated mycorrhizal fungi, thereby increasing their capacity to oxidize and release nutrients (and carbon as a side effect) from organic stocks. This may, for example, explain why elevated CO_2_ levels fails to raise increase belowground carbon stocks in ectomycorrhizal ecosystems, if improved photosynthesis instigates trees to invest more in ectomycorrhizal decomposition (Terrer *et al*., 2021). Finally, this dynamic has potential consequences regarding the development of boreal forest over the nitrogen‐limited Arctic tundra, which could favor carbon loss from soil stocks (Clemmensen *et al*., 2021).

It is important to note that the contrasting nitrogen availability across plots was associated with shifts in dominant tree species and that shifts between pine and spruce dominance also influence fungal community composition and decomposition processes. Therefore, interpretation of these patterns cannot be made solely in terms of nutrient availability, as different tree species are associated with variation in litter quality, root‐derived carbon inputs, host preference for ectomycorrhizal fungi, and Mn availability. In boreal forests, however, these factors are intrinsically linked to natural fertility gradients, where pine commonly dominates nutrient‐poor sites and spruce becomes more abundant in more nutrient‐rich conditions (Shanin *et al*., 2014; Sterkenburg *et al*., 2015; Lukina *et al*., 2019). Therefore, the observed shift in the cellulase producing community from saprotrophic Agaricomycetes toward ascomycetes, as well as the high peroxidase gene expression of ‘ectomycorrhizal decomposers’, under nutrient‐poor conditions, likely reflects the combined influence of several factors associated with nutrient limitation in boreal forest ecosystems, including low pH, different host associations and litter quality.

### Who is doing what?

The ‘decomposer’ trait is not universal among ectomycorrhizal fungal lineages but restricted to a few genera (Zak *et al*., 2019) with certain species being particularly prominent (Lindahl *et al*., 2021; Packard *et al*., 2025). Therefore, genera or species with this capacity should hold a central place in biodiversity conservation effort, as their decline, due to global changes (e.g. wildfire; Pérez‐Izquierdo *et al*., 2021) or human activities (e.g. clear‐cutting; Kyaschenko *et al*., 2017a), could have profound consequences for ecosystems. This emphasizes the importance of more precisely characterizing these functional groups.

*Cortinariaceae* has been repeatedly proposed as a keystone family of ectomycorrhizal fungi with species involved in peroxidase driven decomposition in boreal forest soils (Bödeker *et al*., 2014; Lindahl & Tunlid, 2015; Kyaschenko *et al*., 2017a; Nicolás *et al*., 2019; Lindahl *et al*., 2021; Richy *et al*., 2024; Packard *et al*., 2025). Our findings confirm this role by demonstrating active and ample transcription of class II peroxidase genes by *Cortinariaceae* species under field conditions and at the ecosystem scale. Moreover, we observed other ectomycorrhizal fungi with this ‘decomposer’ trait, albeit with lower shares of the transcripts. For instance, *Russulas* were the second most represented ectomycorrhizal AA2 fungal transcribers, and their GT48 transcripts accounted for a larger share under N‐poor conditions compared to N‐rich. *Hebeloma* species also exhibited AA2 transcripts under N‐poor conditions. Other transcripts matched with *Hysterangium* species, who are since long known to form ectomycorrhizal mats characterized by intense peroxidase activity and extensive decomposition (Entry *et al*., 1991). A role as ‘ectomycorrhizal decomposers’ has already been suggested for some *Hebeloma*, *Russula*, and *Hysterangium* species, as it is known that, like certain *Cortinariaceae* species, they have retained genes encoding oxidative enzymes from saprotrophic ancestors (Bödeker *et al*., 2009; Miyauchi *et al*., 2020). Thus, fungi from these genera may benefit from the support of trees, in terms of root‐derived sugars, to yield the energy required to actively contribute to the oxidation of soil organic matter. Liberation of nitrogen from complex organic matter should enable them not only to proliferate in nitrogen‐limited environments relative to saprotrophs and other ectomycorrhizal fungi but also to be more generous suppliers of nitrogen to their hosts (Jörgensen *et al*., 2025; Packard, 2026). On the contrary, *Piloderma* and *Thelephora* species with no or negligible peroxidase gene expression were hampered under N‐poor conditions, as indicated by a lower share of GT48 transcripts. Although *Piloderma* species have previously been classified among the ‘nitrogen‐miners’ (Argiroff *et al*., 2022; Jörgensen *et al*., 2024), our results challenge this characterization, at least within the scope of our study.

Interestingly, the proportion of GT48 transcripts from *Suillus* fungi was notably high under N‐poor conditions, despite no detectable peroxidase expression from this pine specific fungal genus, which lacks peroxidase genes (Miyauchi *et al*., 2020). Some *Suillus* species may employ a Fenton reaction to oxidize complex organic compounds (Burke & Cairney, 1998; Shah *et al*., 2016), which could explain their efficient transcription of genes related to fungal growth under nitrogen‐limited conditions. Thus, a capacity to produce class II peroxidase – and consequently to release nitrogen from soil organic matter – has only been confirmed for a restricted group of ectomycorrhizal fungi (i.e. *Cortinariaceae*, *Russula*, *Hebeloma*, and *Hysterangium*, in the studied boreal ecosystem) and should not be generalized to all ectomycorrhizal fungi (Pellitier & Zak, 2017), nor can it be inferred solely based on genomic information (Barbi *et al*., 2019).

### Targeted metatranscriptomics: a tool for hypothesis‐driven science

In general, progress in a research field is often accompanied by a shift from observation‐based to hypothesis‐driven science, where more precise hypotheses are constructed using previously accumulated knowledge. As highlighted by Prosser (2020), microbial ecology is currently in a transitional phase and should not be limited to addressing technical challenges but focus on the scientific questions and hypothesis. For this study, we developed a pipeline for targeted metatranscriptomics – that is instead of assembling whole metatranscriptomes, we extracted reads from gene families of interest, according to our scientific questions, and proceeded to specific assembly, curation and taxonomic affiliation of the contigs per gene family. Whereas we intentionally focused on a limited number of functional markers associated with specific processes relevant to our hypotheses, we acknowledge that organic matter decomposition involves a broader diversity of oxidases, oxidative mechanisms (e.g. lytic polysaccharide monooxygenases, Fenton reactions), and other hydrolases than those targeted here. Expanding this approach to additional CAZymes would provide a more comprehensive representation of decomposition.

The motivations behind our focused approach were both technical and conceptual. We did not attempt to cover the full range of fungal decay processes or to reconstruct the full decomposition machinery but rather to assess whether selected functional markers could provide ecologically informative insights into specific and well‐described biochemical components of decomposition and their taxonomic origin in a single boreal forest site. While metatranscriptome analyses can give a comprehensive view of metabolic pathways and a wide appreciation of the ongoing process directly in the field (Auer *et al*., 2024), the amount and complexity of the data call for highly automated gene annotation, which is sensitive to errors in incomplete and poorly curated reference databases. In this context, our targeted approach is less demanding in terms of computational resources and enables detailed and manual analysis of the assembled sequences in a precision‐oriented effort rather than through mass annotation. More targeted and detailed analyses may be considered as a complementary approach within the emerging field of metatranscriptomics and a useful tool for hypothesis‐driven science.

Regarding the gene families tested in this study (AA2, GH6, GT48), the high median coverage of the assembled contigs (6–10× for all contigs, 26–105× for the most expressed contigs), combined with the inclusion of non‐fungal sequences (e.g. bacterial GH6, plant L‐ascorbate peroxidases, plant callose synthases, cytochrome C peroxidases), suggests that we successfully captured the entire metatranscriptomes of the targeted fungal gene families. In addition, we were able to assemble full‐length contigs, and some of the most expressed ones had a perfect match (e.g. AA2_54 with *Mycena sanginolenta*) or almost perfect matches (e.g. AA2_104 with *Rhodocollybia butyracea*, AA2_27 with *Cortinarius ominosus*) to reference sequences, enabling identification of transcribing species (Table S2). Furthermore, GT48 expression may provide a useful indicator of active fungal biomass, as it largely reflects ITS‐based community composition and thus offers cross‐validation between the two approaches. Finally, it is important to note that targeted metatranscriptomics depends heavily on high‐quality reference sequences and databases. These are essential for HMMs ‘probe’ design to capture reads from specific gene families without discrimination and ensuring accurate taxonomic affiliation.

### Conclusion

Under severe nitrogen limitation in boreal forest, certain ectomycorrhizal fungi, which can be characterized as ‘ectomycorrhizal decomposers’, increased community‐level expression of genes coding for class II peroxidases, potentially to ensure mobilization of tightly bound organic nitrogen (in environments with low Mn availability). This highlights the importance of keystone ectomycorrhizal fungal genera – that is *Cortinariaceae* and, to a lesser extent *Russula*, *Hebeloma*, and *Hysterangium* – in the functioning of boreal forests.

## Competing interests

None declared.

## Author contributions

FB and BDL designed research. FB and BDL participated in the fieldwork. FB performed lab work. FB, UM, DS and BDL were involved in bioinformatic pipeline development. TN provided essential data. FB and BDL analyzed the data. FB and BDL wrote the manuscript. All the authors reviewed and contributed to the final version of the manuscript.

## Disclaimer

The New Phytologist Foundation remains neutral with regard to jurisdictional claims in maps and in any institutional affiliations.

## Supporting information


**Fig. S1** Phylogenetic trees of targeted gene families.

**Table S1** Site characteristics and vegetation.

**Table S2** Class II peroxidases summary table (AA2).

**Table S3** Cellobiohydrolases summary table (GH6).

**Table S4** Glucan synthases summary table (GT48).

**Table S5** β‐tubulin summary table (TUB).

**Table S6** Fungal OTUs (ITS).

**Table S7** Origins of the metatranscriptome reads.
Please note: Wiley is not responsible for the content or functionality of any Supporting Information supplied by the authors. Any queries (other than missing material) should be directed to the *New Phytologist* Central Office.

## Data Availability

Sequence data generated during the current study are deposited in SRA (BioProject PRJNA412619, https://www.ncbi.nlm.nih.gov/bioproject/?term=PRJNA412619). Sequence data for *Cortinarius* additional data of *Cortinarius alces*, *C. bovarius*, *C. glandicolor*, and *C. neofurvolaesus* are deposited in European Nucleotide Archive (Study ID PRJEB49625), as well as in SRA for C. *ominosus* (BioProject PRJNA791499) and *C. caperatus* (BioProject PRJNA1277778).
